# Assessment ecological risk of heavy metal caused by high-intensity land reclamation in Bohai Bay, China

**DOI:** 10.1371/journal.pone.0175627

**Published:** 2017-04-19

**Authors:** Gaoru Zhu, Zhenglei Xie, Tuoyu Li, Zongwen Ma, Xuegong Xu

**Affiliations:** 1 Transport Planning and Research Institute, Ministry of Transport of the People's Republic of China, Beijing, China; 2 Key Laboratory for Earth Surface Processes (Ministry of Education), College of Urban and Environmental Sciences, Peking University, Beijing, China; 3 College Geography & Environmental, Jiangxi Normal University, Nanchang, Jiangxi, China; 4 Editorial Department of Journal of Capital Normal University, Capital Normal University, Beijing, China; 5 China Science and Technology Exchange Center, Ministry of Science and Technology, Beijing, China; Beijing Normal University, CHINA

## Abstract

The article examines the detailed spatial and temporal distributions of coastal reclamation in the northwest coast of Bohai Bay experiencing rapid coastal reclamation in China from 1974 to 2010 in annual intervals. Moreover, soil elements properties and spatial distribution in reclaimed area and inform the future coastal ecosystems management was also analyzed. The results shows that 910.7 km^2^ of coastal wetlands have been reclaimed and conversed to industrial land during the past 36 years. It covers intertidal beach, shallow sea and island with a percentage of 76.0%, 23.5% and 0.5%, respectively. The average concentration of Mn is 686.91mg/kg and the order of concentration of heavy metal are Cr>Zn>As>Ni>Cu>Pb>Cd>Hg. We used the "space for time substitution" method to test the soil properties changes after reclamation. The potential ecological risk of heavy metal is in low level and the risk of Cd and As is relatively higher. The ecosystem-based coastal protection and management are urgent to support sustainable coastal ecosystems in Bohai bay in the future.

## Introduction

Coastal wetlands are an interface of the geosphere, hydrosphere, atmosphere, and biosphere that closely link marine and terrestrial ecosystems, and populations and economic activities are often highly concentrated in these zones [1,2]. Coastal wetlands provide a significant number of ecological services and play a fundamental role in guaranteeing ecological security and sustainable development in coastal zones in China [3]. Coastal wetland ecosystems have suffered pressure from extensive wetland reclamation activities in recent decades [2,4]. Approximately 70% of large Chinese cities are located in coastal zones and coastal development has played a leading role in the national economy, contributing 60.8% to its gross domestic product (GDP) and supporting 43.5% of the population [5,6]. China has a long history of coastal reclamation and has continuously decreased coastal ecosystems at a large scale to transform land for agricultural, urban, and industrial uses since the PR China was founded [7]. Approximately 88,372.35 ha of coastal areas have been reclaimed across China, at an average area annual rate of 9819.15 ha from 2002–2010 since the Marine Utilization Management Method was launched [8,9]. Over 70% of coastal reclamation occurred in the northern coastal region of China, and approximately 35% of the reclamation occurred in Bohai Bay [5,10].

Coastal reclamation, which is a prevailing approach to land acquisition and to meeting the growing demands of agriculture, mariculture, industrial development, and urban development, is among the most widespread threats to wetlands in China [8,11]. Tian et al. (2016) [6] reported how coastal reclamation has resulted in rapid losses of vegetated coastal wetlands and caused related environmental impacts. Many countries, including those in developed countries, such as Holland, Japan [12] and Korea [13], and in developing countries, such as China [6] and Indonesia, which aims to reclaim 10,000 ha from the sea, have anticipated large-scale reclamation since the 16th century [14,15]. Large-scale coastal reclamation and infrastructure projects have met the needs of population growth and have brought substantial economic benefits; however, they have also introduced a continuous loss of coastal ecosystem functions and services, along with fishermen’s livelihoods; of particular concern is the increasing risk of disaster related to extreme climate events [5,6]. The direct effects of coastal reclamation activities on natural attributes include changes to the natural coastline length and sea area, which cause the resistance ability decline to defend the natural disaster [11]. The coastal reclamation increases the vulnerability of human settlements to climate change because coastal wetlands act as natural buffers to reduce wave action and shoreline erosion and to attenuate storm surges [16]. Coastal reclamation alters important physical, chemical and biological processes of intertidal habitats and strongly impacts community structure, inter-habitat linkages and ecosystem services while also driving habitat loss [4]. Cui et al. (2016) [10] used several indices extracted from SPOT satellite images and found that while the shoreline length, wetland area, and fractal dimension generally increased under natural conditions, reclamation activities made it more difficult to predict changes. Feng et al. (2014) [17] analysed and assessed the coastal reclamation suitability at the system level in the Jiangsu coastal zone.

Many studies have been carried out to investigate the environmental characteristics and biotic community responses to both habitat loss and sediment burial resulting from marine reclamation [18]. Zhu et al. [19] examined how reclamation affected the shoreline morphology between 1987 and 2012 in two regions of the Yangtze River Estuary in China, where reclamations have been expansive. Reclamation, which decreases soil carbon accumulation and storage capabilities, is one of the key factors that drives soil carbon loss in wetlands [15]. Xiao et al. [20] found that soil type and aggregate distribution were important factors controlling heavy metal concentration and fractionation in the Yellow River Delta wetland soil and suggested that oil exploitation and wetland restoration activities may influence the retention characteristics of heavy metals in tidal soils through variation of soil type and aggregate fractions. Zhang et al. [21] collected surface soil from four chronological sequences of wetlands in the Yellow River Delta of China and found an increasing trend for Pb, Cu, and Zn along the wetland-forming chronosequence although their pollution levels were low. As, Cd and Ni were identified as heavy metals of primary concerns in four wetlands, Cr were of moderate concern in older wetlands, and Pb, Cu and Zn should be paid more attention in younger wetlands. Lu et al. [22] collected surface soils at five sampling sites along a 250-m sampling zone perpendicular to a tidal creek in the *T*. *Chinensis* wetland of the Yellow River Delta. The geoaccumulation index indicated that there was no Cu, Pb or Cr pollution at five sampling sites during all sampling periods. Significant amounts of agricultural, domestic and industrial wastes are discharged into rivers, estuaries, and coastal areas, which results in increasing contamination from heavy metals in sediments [1,23,24,25]. Li et al. [26] investigated the heavy metal (Zn, Ni, Cr, Cu, Pb, and Cd) concentrations in coastal wetland sediments in the Pearl River Estuary. The concentrations of Al, Fe, Cr, Cu, Ni, V, Zn, As, Co, and Pb in the surface sediments of Chabahar Bay were studied to assess the degree of heavy metal pollution resulting from natural and anthropogenic sources [27]. Bai et al. [28] collected soil samples in tidal freshwater and salt marshes before and after flow-sediment regulation in the Yellow River Delta of China.

The northwest Bohai Bay, including the Tianjin Binhai New Area and Coafeidian New Area, is experiencing a historically unprecedented wave of urbanization and industrialization and has experienced large-scale land reclamation from sea processes in the past 36 years [9,29]. Many studies have been carried out on ecosystem changes resulting from coastal reclamation during the 1980s and 2010s, including research into coastal wetland dynamics [30] and heavy metal distribution changes [25,31]; however, soil element dynamics and spatial differentiation in newly reclaimed areas remain poorly understood. A need to understand the magnitude of the ecological impacts from coastal reclamation is imperative to guiding coastal management [4]. Thus, improved knowledge of the distribution and influential factors of soil elements and their potential feedbacks to further development strategies across different soil depths is essential to determining whether large-scale coastal reclamation has accelerated ecosystem deterioration. In this research, remote sensing data and surface sediment collection were used to identify the reclamation process from 1974 to 2010 in one-year intervals and to investigate the ecological effects of coastal reclamation areas on Bohai bay, as well as the consequent changes in coastal ecosystems. Therefore, this paper aims to answer the following questions: 1) What were the reclamation processes and trends in Bohai Bay from 1974 to 2010? 2) What are the potential bio-risks of metals in sediments that have resulted from large-scale reclamation? 3) How should coastal management strategies support the sustainable development of resource utilization in Bohai Bay?

## Materials and methods

### Ethics statement

**Fig 1:**
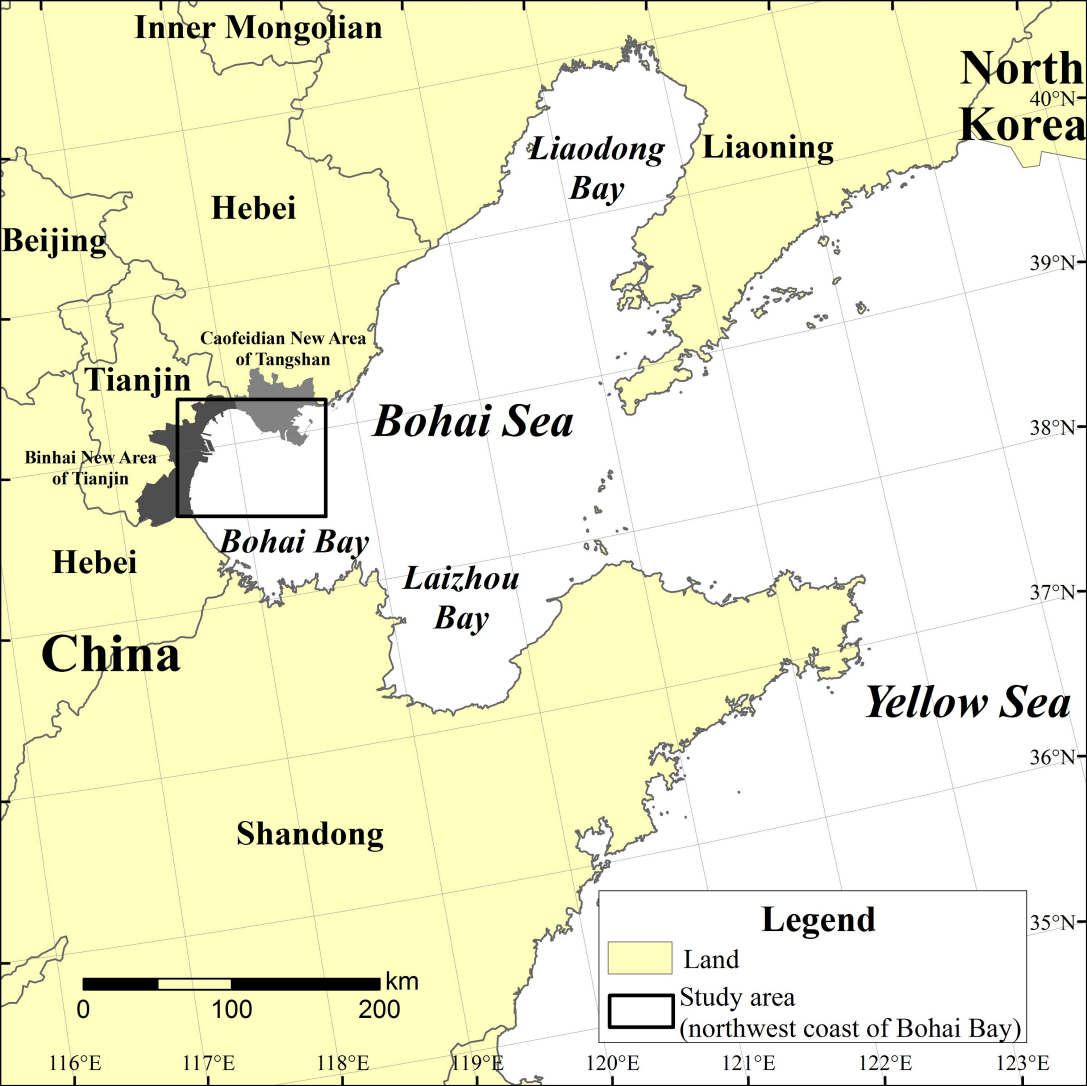
Location of study area of Bohai Bay including Binhai New Area of Tianjin and Caofeidian New Area of Tangshan, Hebei province. The region is experiencing the intensive land reclamation in recent years.

Our study area is located in the Tianjin coastal area, which is owned by the Chinese government. This study did not involve endangered or protected species and no specific permissions were required for the locations/activities in this study. The specific locations in the present study are shown in Fig 1.

### Study area

The study areas is located at 117°4′33″~119°18′31″E,37°33′40″~39°39′31″N and covers an areas of 22,133 km^2^, while the Binhai New Area in Tianjin and Caofeidian New Area in Tangshan Hebei province are the key areas of rapid urban sprawl and large-scale reclamation (Fig 1). The Bohai Bay is a mudflat plain with an elevation range of 55~-10 m above sea level, with a slope of 0.1%~0.6%. The sediments in Bohai Bay are from the river and form the largest coastal bank mudflat. The area is located in the north-temperate zone and belongs to a semi-humid continental climate in the warm temperate zone. The average temperature is 11°C and the region experiences 600~900 mm of precipitation per year. The runoff in different seasons has an obvious range and has exhibited a decreasing tendency in recent years. The natural vegetation is herbaceous plants and shrubs with high salt-tolerance ability. The typical wetland vegetation includes *Suaeda salsa*, *Suaeda glauca*, *Phragmites australis*, *Imperata cylindrical*, *and Tamarix chinensis*. The forest vegetation includes broad-leaved deciduous forests, such as *Pyrus betulaefolia and Fraxinus chinensis*.

Bohai Bay is a typical semi-enclosed coastal sea with mean depth of 12.5 m [11]. The study area has a lower slope and abundant sediments that are beneficial for land reclamation. Approximately 35% of the reclamation has occurred in Bohai Bay. From 1996 to 2007, the reclaimed area was 551 km^2^, the mudflat area decreased to 718 km^2^, and the shoreline decreased by 260 km, and the Bohai Bay, including the Caofeidian New Area, has occupied a sea area of 310 km^2^. Approximately 307 km^2^ of coastal wetland has been reclaimed in the Binhai New Area over the past 10 years, especially between 2010 to 2014 when the reclaimed area reached 161 km^2^. The northwest Bohai Bay includes Tianjin Binhai New Area, the Fengnan District, and the Caofeidian New Area, and these are the largest artificial reclamation areas planned in the 11^th^-Five-Plan of China. The Tianjin Binhai New Area has turned a desolate coast into a modern metropolitan area. The Caofeidian New Area has been one of the national comprehensive strategies in development in 2010 and is promoting the construction of the Caofeidian International Eco-city. These areas have the largest integrated port and coral transportation harbour, serve as an important iron and steel base, petroleum base, and salt base. The study area had a population of 4.367 million in 2008, with an off-farm population of 1.53 million. The Gross Domestic Production (GDP) in 2008 was 443.1 billion RMB and the per Capita GDP was 101,000 RMB.

### The coastal reclamation process from 1974 to 2010

Cloud-free Landsat images from 1974 to 2010 at one-year intervals were collected at a 30 m spatial resolution to assess landscape changes during coastal reclamation in the Bohai Bay [32]. The reference data consists of a topographic map from 1981, the national land use database (1986, 1995, 2000), and a land use status map (1997–2010). Moreover, a field investigation launched in September 2009 and July 2010 collected 242 samples of location, vegetation, and soil conditions using a GPS portal. Visual interpretation of Landsat images from 1974 to 2010 was applied to identify the annual reclamation process with the help of DEM data, an administrative zone map, the mudflat boundary, and water depth line data. Owing to the differences in image quality, individual features were established for each image during the image interpretation. The maximum livelihood method of supervised classification was applied to discriminate the area features with relatively high spectrum traits differentiation, such as paddy fields, dryland, forest land, grasslands, built-up areas, water bodies, and bare land. Approximately 100–120 training zones that were evenly distributed in each image were selected and patch areas less than 1 hm^2^ were removed; the visual interpretation was finished in the ArcGIS 9.3 platform. Approximately 1200 random points were selected to compare the interpretation results and reference information based on the Kappa coefficient. The overall interpretation accuracy in 1979, 1989, 1999, and 2008 was 83.3%, 85.9%, 88.8%, and 87.5%, respectively. Specific information from the interpretation process can be referenced from Zhu and Xu (2012) [32].

Based on "space for time", the environmental change process in the coastal reclamation area was explored [32]. The geography element maintained rich time-series change information. It is significance to deduce the surface land feature changes in the time-series with the help of the spatial differentiation of surface features. This study analysed soil trait differentiation during different reclamation periods in reclaimed areas and applied the method of "space for time" to investigate the environmental change on a decadal scale with the interpreted remote sensing data.

### Soil property changes caused by coastal reclamation

**Fig 2:**
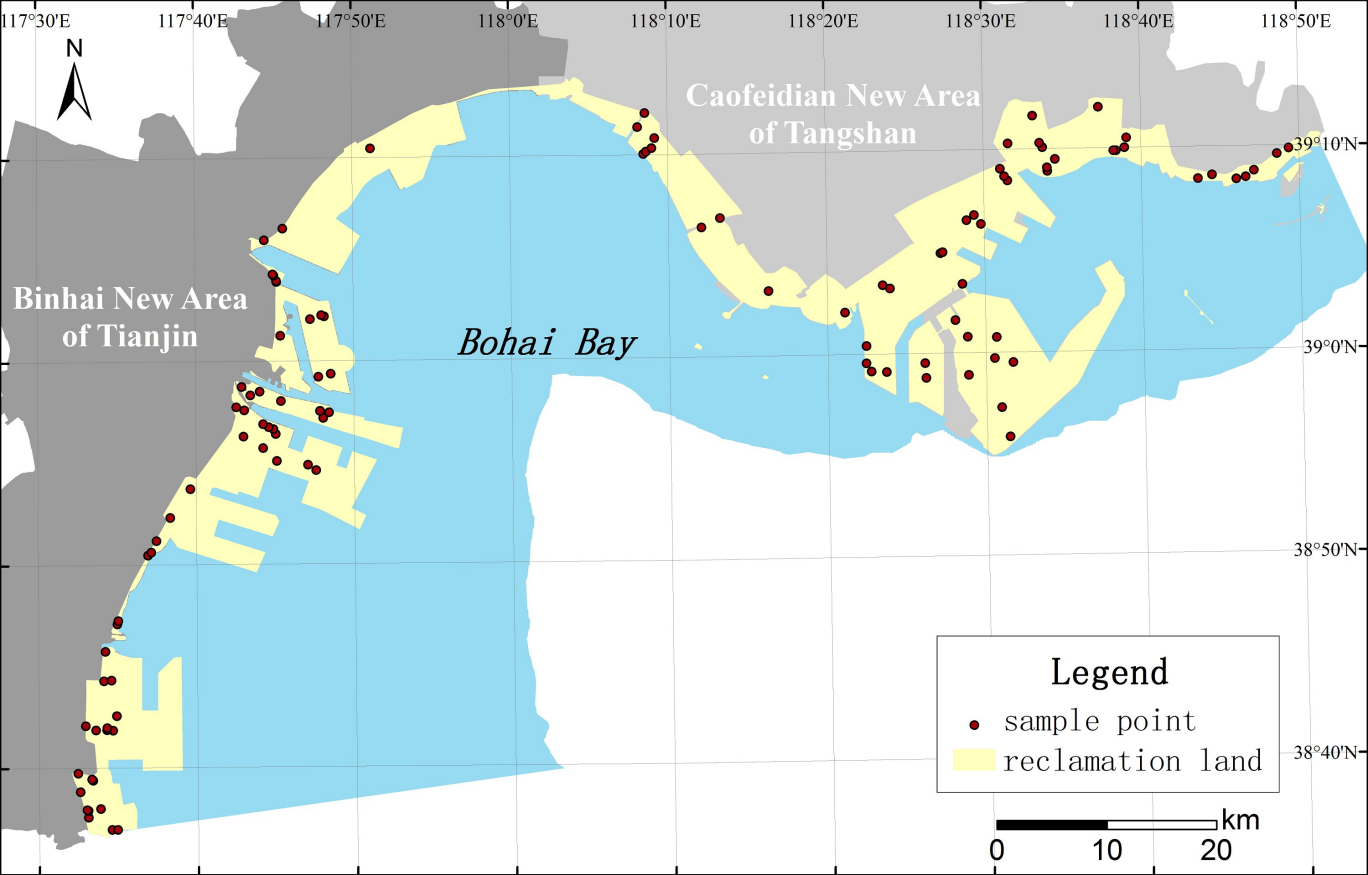
Soil sample point in northwest of Bohai Bay. During 2010 and 2011, approximately 112 soil samples were obtained at depth intervals of 0~25 cm, 25~50 cn. These samples points is located in the reclamation area in Bohai Bay.

The integrated environment survey and sample collection were carried out to understand the soil surface properties in newly reclaimed coastal areas since 1974 in northwest Bohai Bay and to analyse the spatio-temporal changes in soil properties. A sample points survey was applied using a stratified random sampling method and maintained different land use types in each period with samples (Fig 2). During 2010 and 2011, approximately 112 soil samples were obtained at depth intervals of 0~25 cm (surface layer), 25~50 cm (below layer), and the replicated samples were mixed together at each location to form a composite samples. All of the soil samples were placed in polyethylene bags and brought to the laboratory, where they were air-dried at room temperature for three weeks. The air-dried soil was grounded and passed through a 2-mm nylon sieve to remove coarse debris.

The spatial location was located using a GPS portal, a ring sampler was used to detect volume weight, and an oven-drying method was used to detect the water content; the organic matter was tested with a total organic carbon analyser, while heavy metal elements, such as Pb, Cr, Ni, Zn, As, Cd, and Mn, were determined using the ICP-AES method. Specific analysis can be referenced from Xie et al. (2014) [33]. Mathematical statistics analysis and the relationship between soil properties and heavy metal concentrations involved SPSS 18 software, while Kriging and inverse distance weighted (IDW) spatial interpolation are used to identify spatial differentiation of soil elements. A correlation analysis of different heavy metals was used to determine the source of heavy metals, the intensity of human activities, and the factors controlling concentration changes. The potential ecological risk parameter is an effective method for assessing heavy metal concentrations and its ecosystem effects [34,35]. The formula is as follows:

$$C_{f}^{i}=\frac{C^{i}}{C_{n}^{i}}\;\;C_{d}=\sum_{i=1}^{7}C_{f}^{i}\;E^{i}=T^{i}\times C_{f}^{i}\;RI=\sum_{i=1}^{7}E_{r}^{i}$$

where $C_{f}^{i}$ is the pollution index of heavy metal i in surface soil, *C*^*i*^ is the concentration value of heavy metal i, $C_{n}^{i}$ is the background reference value of heavy metal i, *C*_*d*_ is the integrated pollution index of heavy metal i, *T*^*i*^ is the toxicity response parameter of heavy metal i and can demonstrate the toxicity of heavy metals and the sensitivity extent of an organism to heavy metal pollution, and *E*^*i*^ is the potential ecological risk value of heavy metal i. RI is the integrated potential ecological risk parameter of heavy metal elements. The background reference values of Cu, Pb, Cr, Ni, Zn, As and Cd in Tianjin are 28.8, 21.0, 84.2, 33.3, 79.3, 7.4, and 0.09 mg/kg and the toxicity response coefficient of a heavy metal element are 5, 5,2,5,1,10,30, respectively. The potential ecological risk parameter not only reflects the effect of a single heavy metal upon environment change but also demonstrates the integrated influential extent of heavy metal factors upon surrounding environments. *E*^*i*^ and RI are the indexes representing the potential heavy metal ecological effects.

## Results

### The coastal reclamation process on the northwest coast of Bohai Bay

**Fig 3:**
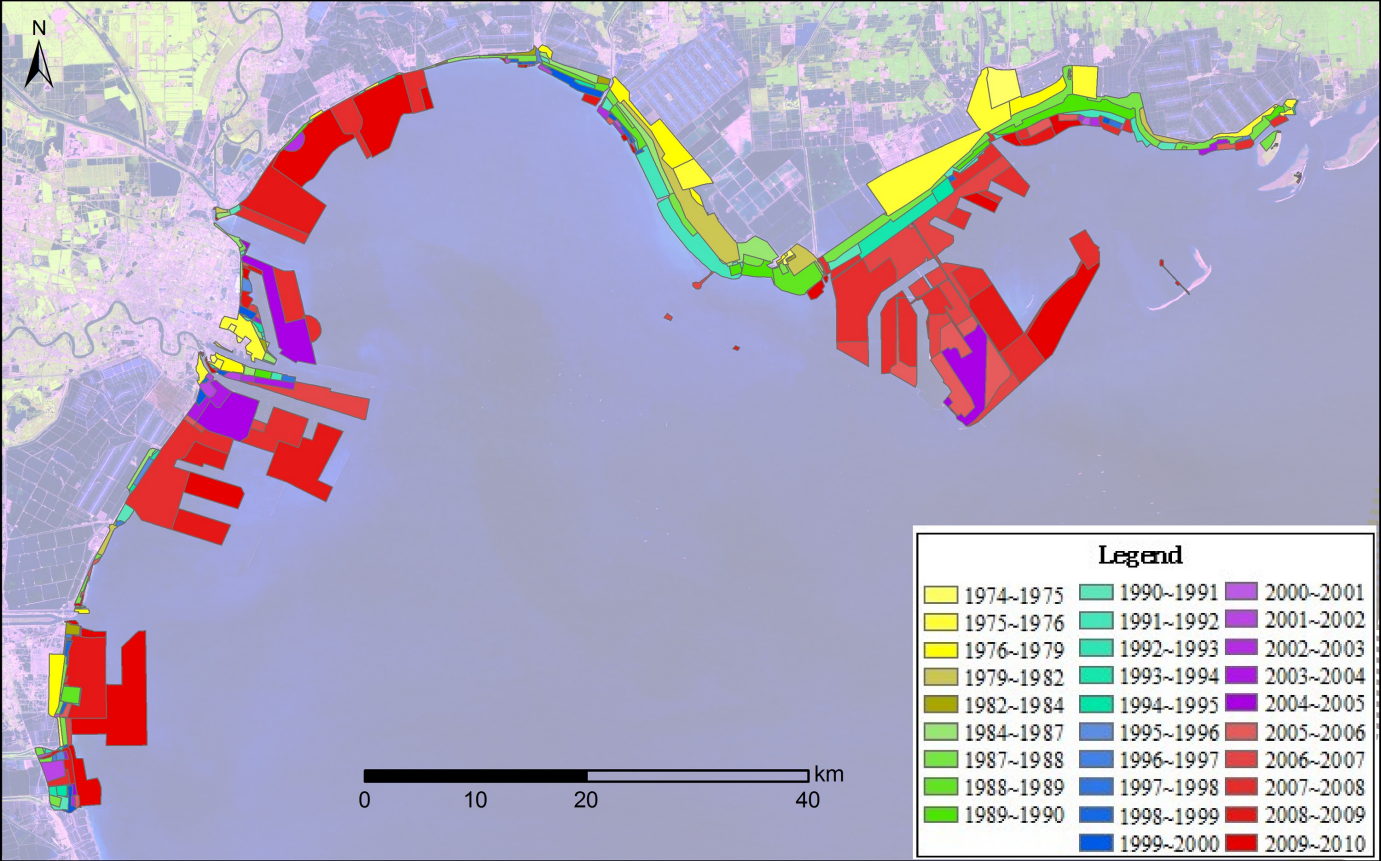
The annual reclamation process in the northwest coast of Bohai Bay during 1974 to 2010. The reclamation process is illustrated through visual interpretation of coastline, mudflat, and offshore boundaries and approximately 901.7 km2 of coastal area was reclaimed during the period.

The coastal reclamation process on the northwest coast of Bohai Bay from 1974–2010 is illustrated in Fig 3 through visual interpretation of the coastline, mudflat and offshore boundaries. The coastal reclamation process could represent human activity intensities; approximately 901.7 km^2^ of coastal area was reclaimed between 1974–2010 and led to the disappearance of 56.9% of mudflat areas, 26.7% of island areas and 9.3% of offshore areas.

### The soil physical and chemical properties in different reclamation areas

The volume weight of the sub-surface layer was larger than that of the surface layer, indicating that the surface layer is loose and has a relationship with soil gravity subsidence. The standard deviation of the volume weight of the surface layer is relatively high and is vulnerable to external disturbances. The volume weight ranged between 0.92~1.73 g/cm^3^, with an average value of 1.29 g/cm^3^, and a standard deviation of 0.14 g/cm^3^ (Table 1). The volume weight is smaller than that of a natural mudflat (1.41~1.59 g/cm^3^) and is higher than that of vegetation fields (0.97~1.45 g/cm^3^) [36]. The vertical distribution pattern of the soil water content indicates that it will increase along the soil depth and demonstrates that the groundwater table is high. The water content ratio is between 4.08% and 60.91%, and the average value is 24.48%. The water content is relatively lower compared with natural salt lands (36.46%~40.89%) and newly reclaimed lands, whereas the water content of the Binhai New Area is lower than that in the Caofeidian New Area of Hebei province.

**Table 1:** Statistics properties of soil properties in reclaimed land.

	Volume weight (g/cm^3^)			Water content (%)			pH			Saltness (g/kg)			SOM(g/kg)
	0-25cm	25-50cm	mean	0-25cm	25-50cm	mean	0-25cm	25-50cm	mean	0-25cm	25-50cm	mean	
Number	112	112	112	112	112	112	112	112	112	112	112	112	112
Mean	1.27	1.30	1.29	22.43	26.53	24.48	8.35	8.35	8.35	4.87	4.52	4.69	14.78
Min	0.90	0.93	0.92	2.30	5.17	4.08	7.83	7.73	7.88	0.00	0.00	0.05	0.60
Max	1.71	1.79	1.73	59.46	62.37	60.91	9.78	11.13	10.01	22.40	23.20	22.80	96.95
Sd	0.17	0.15	0.14	10.60	10.05	9.52	0.37	0.43	0.36	4.05	3.56	3.57	13.65
CV	13.36	11.25	11.02	47.25	37.89	38.90	4.42	5.10	4.36	83.13	78.73	76.18	92.40
Skewness	0.62	0.84	0.63	0.75	0.65	0.64	1.79	3.27	2.14	1.51	1.97	1.62	3.77
Kurtosis	0.43	1.67	1.21	0.67	1.35	1.27	4.17	16.79	6.06	3.93	6.68	5.23	19.20

CV:Coefficient of variation(%)

Sd: Standard deviation

The soil pH average value, saline degree, and organic matter are demonstrated in Table 1. The average value of the pH in the two soil layers (0~25 cm, 25~50 cm) is 8.35, whereas the range of below layers is relatively larger with a high variation coefficient. The pH ranged between 7.88~10.01, and 72.7% of the samples were in the range of 8~8.5 (Table 2). The pH in the Tianjin Binhai New Area was 8~9.5, while the value in the Caofeidian New Area was 7.5~8.5. The soil salinity in the surface layer was higher than that in the sub-surface layer because of high evaporation. The sampling was performed in summer and salinity will ascend along with soil water in the surface layers. The average saline values in the Tianjin Binhai New Area and the Caofeidian New Area were 4.67 g/kg and 4.71 g/kg, respectively. The average value of soil organic matter (SOM) was 9.25 g/kg, demonstrating the defection deposition of SOM on reclaimed land, whereas the surface layer of SOM was higher than that in the sub-surface layer. The variation coefficients of the surface layer were relatively higher. The average SOM concentration in the Tianjin Binhai New Area was 10.5 g/kg, whereas it was 6.5 g/kg in the Caofeidian New Area of Tangshan.

**Table 2:** The correlation coefficient matrix between different soil elements.

	**The weight volume**	**Water content**	**pH**	**Salinity**	**SOM**
The volume weight	1				
Water content	-0.248**	1			
pH	0.231*	-0.292*	1		
Salinity	-0.265**	0.471**	-0.458**	1	
SOM	-0.225*	-0.073	-0.145	0.155	1

**p<0.01.

*p<0.05.

The volume weight was the basis for other soil properties and is positive with the pH, whereas it has a negative relationship with other parameters. The water content has a negative correlation with pH and a positive correlation with saline, indicating that the saline is from seawater or intruded underground salt water. The pH value was positively correlated with the volume weight and had a negative correlation with the water content and salinity, representing how the pH is affected by the above factors (Table 2). There was a negative correlation between SOM and volume weight.

**Fig 4:**
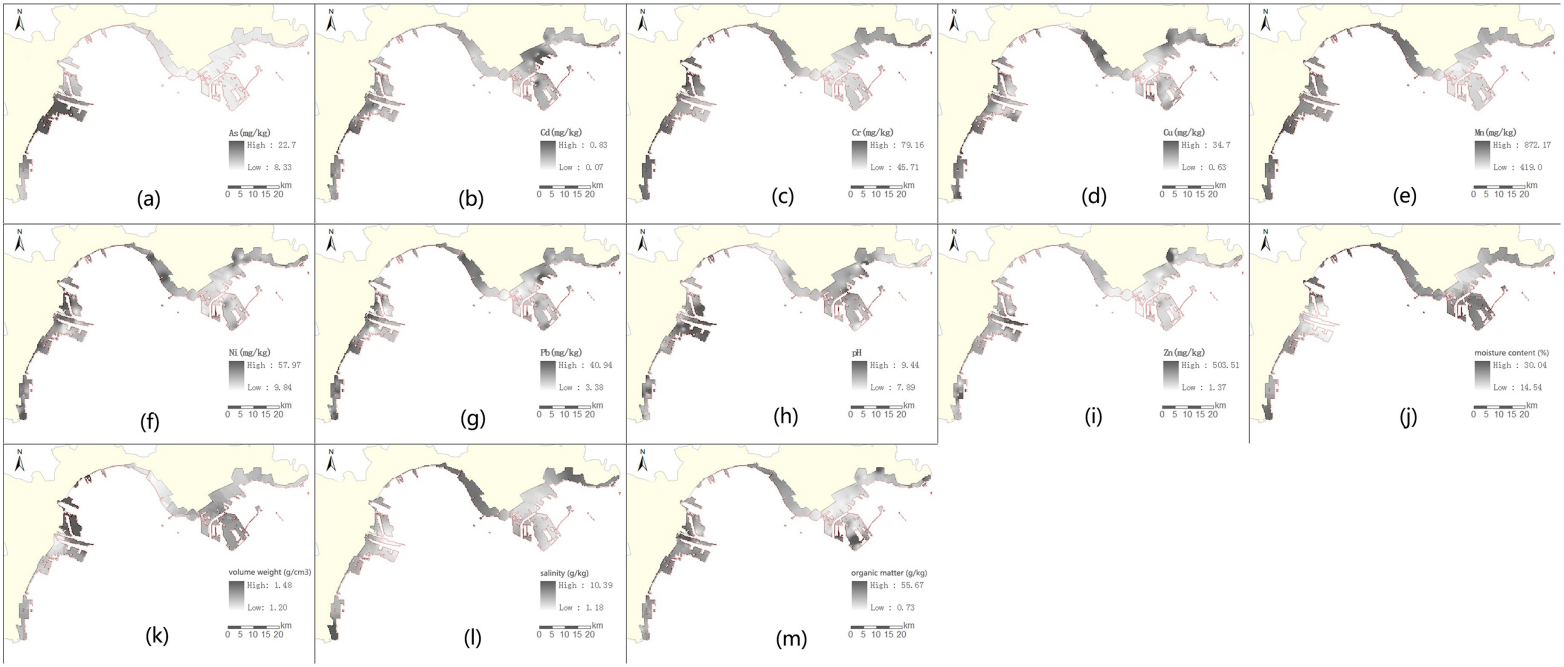
The spatial interpolation of soil properties using Kriginig and IDW spatial interpolation method in the reclamation area. The soil properties includes As, Cd, Cr, Cu, Mn, Ni, Pb, pH, Zn, moisture content (%),volume weight (g/cm^3^),salinity (g/kg), and organic matter (g/kg).

To identify the spatial variation of the soil properties, a Kriging and IDW spatial interpolation method were applied to investigate the spatial distribution of soil elements in the reclamation area (Fig 4). The maximum value of the volume weight was located in Dongjiang, the value of Caofeidian harbour area and the industrial zones were second highest. The soil was sandy, with plenty of mineral substances, a high water content, and saline in the area. Saltwater aquacultural land was located in the harbour area and in the industrial areas of the newly reclaimed Caofeidian New Area, whereas the pH value in the Binhai New Area was larger than that in the Caofeidian New Area. The salinity degree in saline and saltwater aquaculture was larger in this area. The salinity in the newly reclaimed area was lower owing to the poor retention of sandy soil to salinity. The heavy metal elements of Pb, Cr, Ni, Cd, and Mn were high, with a long history of reclamation, whereas Zn was enriched in the Nanjiang portal area and the Caofeidian New Area. A high value of As was located in Nanjiang port and in the industrial zones. The soil factors on the reclaimed land of Bohai Bay’s northwest coast had an obvious spatial differentiation, which was caused by natural and human activities.

### Risk assessment of heavy metal concentration in the reclamation area

The mean concentration and the background value of heavy metals were demonstrated in Table 3. The average concentration of Mn is 686.91 mg/kg and the enrichments decreased along the following order: Cr>Zn>As>Ni>Cu>Pb>Cd>Hg. The range of Cu concentrations are 2.43~40.53 mg/kg, with an average value of 20.31 mg/kg. Approximately 20.5% of the samples exceeded the Tianjin soil background value and 31.25% of the samples exceeded the Bohai Bay surface sediment background value. The average concentration of Cu in the Binhai New Area was 22.20 mg/kg and was higher than that of 3.92 mg/kg in the Caofeidian New Area. The range of Pb was 2.76~65.04 mg/kg, with an average value of 20.96 mg/kg. Approximately 47.3% and 42.8% of Pb concentrations were higher than that of the Tianjin soil background value and the Bohai bay sediment background value, respectively. The Pb average value in the Binhai New Area was 21.74 mg/kg and was slightly higher than that of 21.6 mg/kg in the Caofeidian New Area. The concentration of Cr range from 30.52~204.79 mg/kg, with an average value of 65.77 mg/kg. Approximately 8.2% and 25.8% of the Cr sample concentrations were higher than that of the Tianjin soil background and Bohai Bay sediment background values. The Cr concentration in the Binhai New Area was 71.39 mg/kg and was higher than that of 60.27 mg/kg in the Caofeidian New Area. The concentration range of Ni was 9.53~58.41 mg/kg, with average value of 27.83 mg/kg. Moreover, approximately 29.9% of all of the samples concentrations of Ni were higher than the Tianjin soil background value and the Bohai Bay sediment background value. The average concentration of Ni in the Binhai New Area are 31.11 mg/kg and are higher than that of 24.61 mg/kg in the Caofeidian New Area.

**Table 3:** Content statistics of heavy metal in reclaimed land (unit: mg/kg).

	Cu	Pb	Cr	Ni	Zn	As	Cd	Mn	Hg
Number	112	112	97	97	97	97	97	97	15
Mean	20.31	20.96	65.77	27.83	63.66	13.23	0.142	686.91	0.043
Min	2.43	2.76	30.52	9.53	1.25	4.93	0.040	307.81	0.005
Max	40.53	65.04	204.79	58.41	1285.57	34.80	2.342	1146.16	0.208
Standard deviation	8.80	6.68	21.56	10.49	147.98	6.15	0.23	210.48	0.05
VCoeff(%)	43.33	31.86	32.77	37.71	232.44	46.44	160.22	30.64	119.98
Skewness	-0.06	2.26	2.72	0.18	6.88	1.53	9.57	0.11	2.60
Kurtosis	-1.00	16.47	17.09	-0.51	52.13	2.02	93.33	-1.09	8.10
Tianjin sediment background	28.8	21	84.2	33.3	79.3	7.4	0.09	686	0.084
China sediment background	22.6	23.6	61	26.9	67.7	9.6	0.097	583	0.065
Bohai Bay sediment background	26	22.4	75	34.4	73.6	15.3	0.15	626	0.065

The concentration range of Zn, with an average value of 63.66 mg/kg, was 1.25~1285.57 mg/kg. Approximately 8.2% and 13.4% of the sample concentrations were higher than that of the Tianjin soil background value and the Bohai Bay sediment background value. The concentration of Zn in the Binhai New Areas was 84.10 mg/kg and was higher than the 43.65 mg/kg value in the Caofeidian New Area. The As concentration ranged from 4.93~34.80 mg/kg and had average value of 13.23 mg/kg. Approximately 69.1% and 24.7% of the samples of As concentration were higher than the Tianjin and Bohai Bay background values. The As concentration in the Binhai New Area was 16.01 mg/kg and was higher than that of 10.51 mg/kg in the Caofeidian New Area. The Cd concentration ranged from 0.04~2.34 mg/kg, and the average value was 0.142 mg/kg. Approximately 86.6% and 16.5% of the samples' Cd concentrations were higher than the Tianjin and Bohai Bay background values. The Cd concentration in the Binhai New Areas was 0.13 mg/kg, and it was 0.154 mg/kg in the Caofeidian New Area. The Mn concentration range was 307.81~1146.16 mg/kg, and the average value was 686.91 mg/kg. Approximately 51.5% and 58.8% of the samples' Mn concentrations were higher than the Tianjin and Bohai Bay background value. The concentration of Mn in the Binhai New Ares was 762.13 mg/kg and 613.22 mg/kg in the Caofeidian New Area. The average value of Hg was 0.043 mg/kg, whereas approximately 6.7% and 13.3% of samples of Hg concentrations were higher than the Tianjin and Bohai Bay background values. The concentrations of Hg were 0.058 mg/kg in the Binhai New Area and 0.012 mg/kg in the Caofeidian New Area.

There was an obvious positive correlation between SOM and heavy metal elements, except for Cd and Hg (Table 4). The volume weight was negatively correlated with Cu, Pb, Mn, Hg, indicating that the good soil structure might accumulate more heavy metals contents. The relationship between the water content, saline and heavy metal concentration had a positive correlation. There was an obvious negative correlation between the pH and Cu, Ni, Mn, implying that the heavy metal elements would easily accumulate in soil. The obvious correlation between Cu and other elements indicated that Cu is easily affected by soil properties. As for the relationship between different heavy metal elements, Table 5 demonstrates that the heavy metal elements have an obvious positive correlation, except for Zn and Cd, implying that they had a high spatial relationship and had high source similarity.

**Table 4:** The correlation coefficient matrix between soil heavy metal and soil properties.

	Cu	Pb	Cr	Ni	Zn	As	Cd	Mn	Hg
VW	-0.404**	-0.301*	-0.034	-0.245	0.055	0.041	-0.039	-0.358**	-0.622*
WC	0.266*	0.079	0.066	0.125	-0.0813	-0.085	-0.016	0.071	0.638*
pH	-0.307**	-0.154	-0.172	-0.323**	-0.033	0.139	-0.039	-0.245*	-0.287
Salinity	0.454**	0.180	0.220*	0.413**	0.044	-0.052	-0.067	0.388**	0.807**
SOM	0.326**	0.299**	0.321**	0.496**	0.097	0.272**	-0.069	0.537**	0.227

** p<0.01

* p<0.05

**Table 5:** The relationship coefficient matrix among different heavy metal element.

	**Cu**	**Pb**	**Cr**	**Ni**	**Zn**	**As**	**Cd**	**Mn**
Cu	1							
Pb	0.593**	1						
Cr	0.667**	0.446**	1					
Ni	0.920**	0.496**	0.798**	1				
Zn	0.172	0.069	0.077	0.178	1			
As	0.373**	0.365**	0.218*	0.277**	0.274**	1		
Cd	0.006	0.774**	0.044	-0.070	0.008	0.219*	1	
Mn	0.924**	0.557**	0.664**	0.913**	0.129	0.410**	-0.010**	1

** p<0.01

* p<0.05

**Fig 5:**
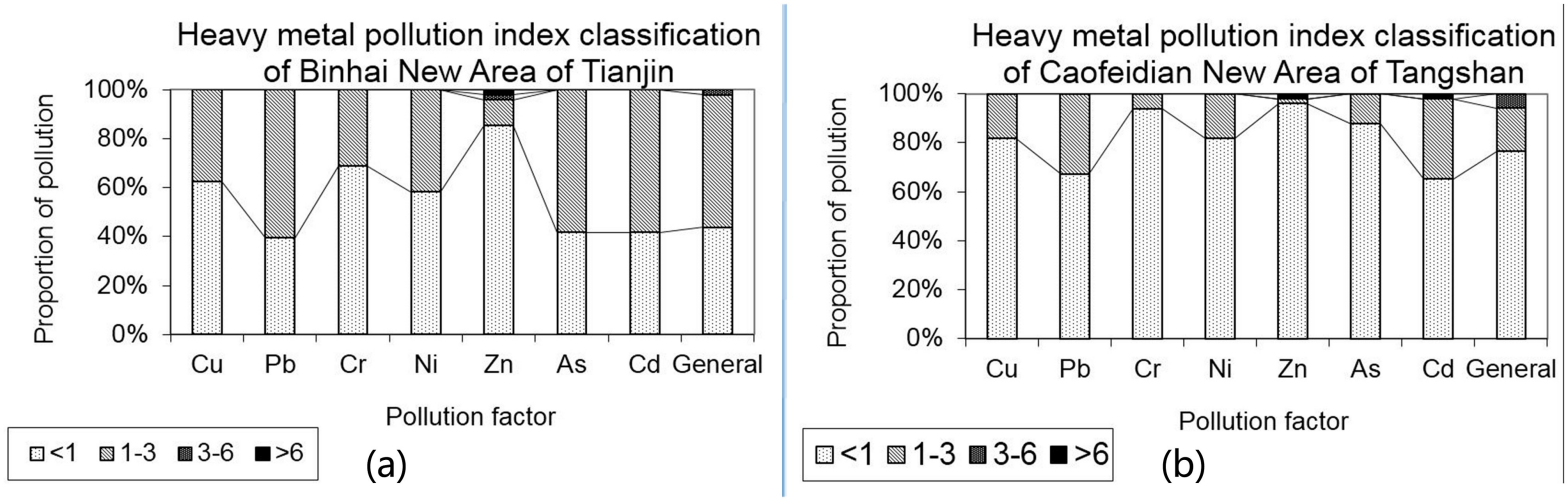
The heavy metal pollution index ration in Binhai New Area and Caofeidian New Area. The heavy metal pollution index exhibited obvious in different areas. The heavy metal elements of Pb, Zn, As, and Cd had a moderate pollution level and other elements were at low pollution levels in Binhai New Areas. The pollution level in the Caofeidian New Area was lower than that in the Binhai New Area.

The heavy metal pollution index exhibited obvious differentiation in different areas. As for the Binhai New Areas, the heavy metal elements of Pb, Zn, As, and Cd had a moderate pollution level and other elements were at low pollution levels; the integrated pollution index was at the moderate level (Table 6; Fig 5). In terms of each element, there were a few samples with Pb, As, Cd concentrations at low pollution levels, whereas Zn concentrations in many samples were found at serious pollution levels. Only the Cd concentration in the Caofeidian New Area was found at a moderate pollution level, and other elements were found at low pollution levels, whereas the integrated pollution level was low. Most of the Cd concentration samples were found at a moderate level. Thus, only Pb, As, and Cd were found at a moderate pollution level, and the pollution level in the Caofeidian New Area was lower than that in the Binhai New Area.

**Table 6:** The average heavy metal single factor and integrated pollution index.

	Cu	Pb	Cr	Ni	Zn	As	Cd	The integrated pollution index
Tianjin Binhai NA	0.87	1.04	0.90	0.92	1.10	1.29	1.08	7.19
Caofeidian NA	0.71	0.95	0.76	0.73	0.57	0.84	1.29	5.85
Study area	0.79	1.00	0.83	0.82	0.83	1.06	1.18	6.51

According to Table 7, there was a relatively obvious differentiation of potential heavy metal ecological risk among difference elements and they had no obvious differentiation in different areas. In term of the Tianjin Binhai New Areas and Caofeidian New Areas, the potential ecological risk index from Cd, As, Pb was relatively high, below a value of 40, and represented a low pollution level. The integrated potential ecological risk index in both areas did not exceed 131. Although the heavy metal pollution level of several samples was relatively high, such as in the Caofeidian New Area, and the integrated potential ecological risk parameter of one sample was 625, representing extremely polluted levels. However, the maximum value of the other samples was only 94 and indicated a low pollution level. Therefore, the heavy metal ecological risk value was relatively low and the ecological risk value in descending order was Cd>As>Pb>Ni>Cu>Cr>Zn.

**Table 7:** The average heavy metal singe factor and integrated potential eco-risk index.

	Cu	Pb	Cr	Ni	Zn	As	Cd	Integrated potential eco-risk index
Tianjin Binhai NA	4.37	5.19	1.79	4.60	1.10	12.86	32.42	62.32
Caofeidian NA	3.53	4.77	1.51	3.64	0.57	8.45	38.62	61.08
Study area	3.95	4.98	1.65	4.11	0.83	10.63	35.55	61.70

**Fig 6:**
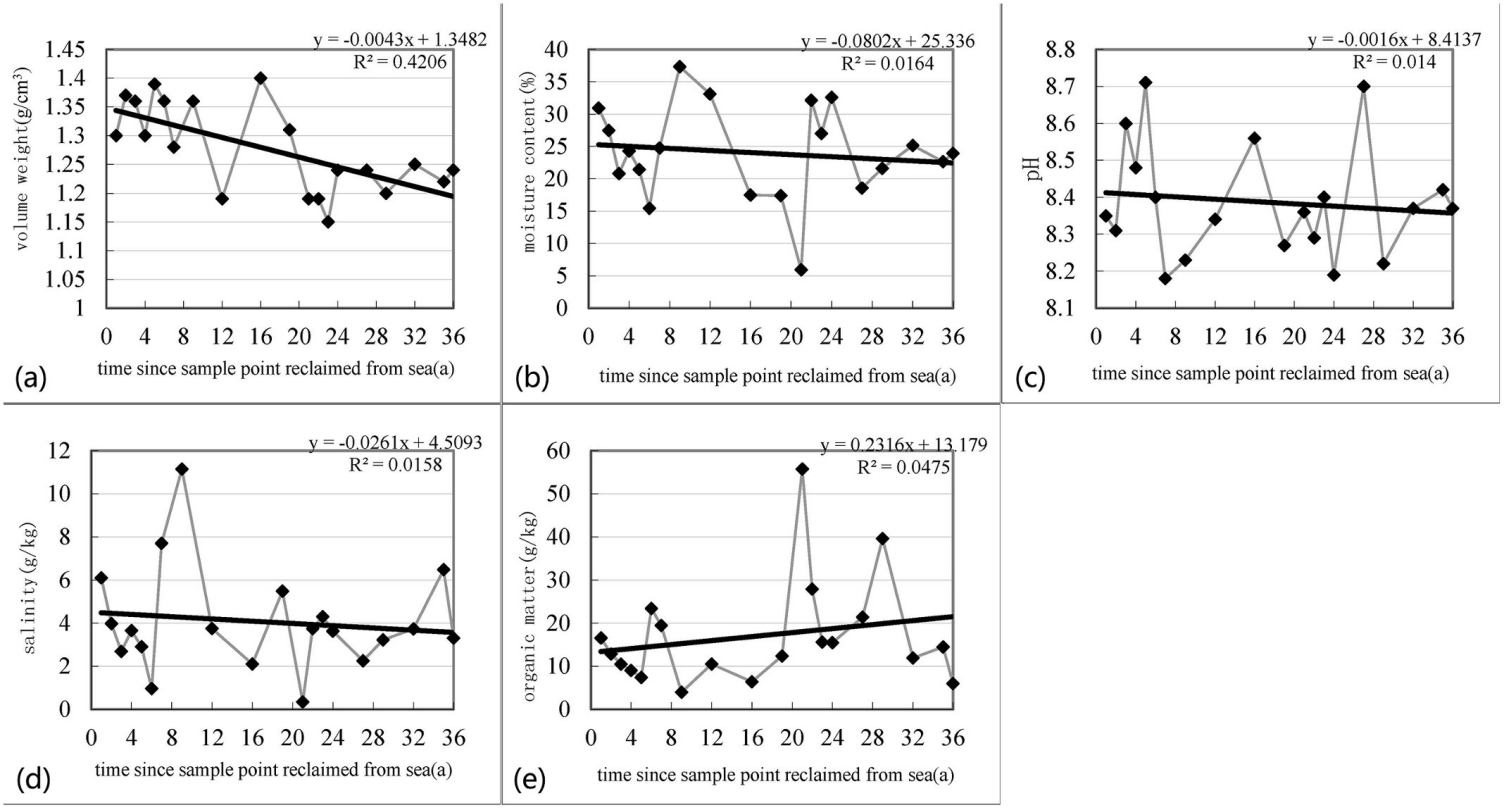
The soil properties change trend along the reclamation sea. The soil volume weight demonstrates a downward trend over 36 years and the soil water content decrease gradually. The pH, and salinity exhibited a downward trend, especially in the first 6 years. The salinity degree in the first year was 6.1 g/kg and it decreased along the reclamation period. The soil organic matter concentration appear increased trend during the reclamation period.

Fig 6 represents the soil volume weight with a downward trend over 36 years (r = 0.65). After the reclamation process was finished, the soil became compacted along from the moisture rate unwatering and from gravity. Moreover, many engineering methods, such as cacuum proloading and loading preconsolidation, will accelerate the process to increase the base loading ability and to make the soil water content and the void ratio decrease. Since the reclamation process was completed, the water content decreased gradually (r = 0.13) along with gravitational influences, and an engineering method was applied, quickly excluding water content. This trend is obviously visible during the first 6 years when the water content decreased by 50%. In the initial years, along with a natural sink and drainage, the soil water content declined quickly and soil became more consolidated. The decreased trend will not be changed.

The degree of soil salinity exhibited a downward trend in the past 36 years (r = 0.13), especially in the first 6 years. The sand used for reclamation had a relatively high salinity degree and would reach 20–50 g/kg. Since the reclamation process was completed, the water content loss would increase the salinity degree, whereas precipitation or irrigation would make the soil salinity degree decrease. The salinity degree in the first year was 6.1 g/kg and it decreased along the reclamation period and reached a non-salinization level (<1 g/kg) in the 6th year, which was suitable to construct infrastructure. The soil pH exhibited a downward trend in the reclamation period of 36 years and it demonstrated an obvious increasing trend. The pH value of coastal saline soil during the initial period increased, and it lost saline content because of soil leaching and increased the HCO^3-^ concentration of the soil solution desalting alkalization, and leading to an increase in the pH value. With vegetation growth and succession development over a long period, the vegetation biomass would increase and produce more litter, and abundant litter decomposition would produce more CO_2_ and organic acid, decreasing the pH soil value. The SOM concentration would increase during the reclamation period and decrease from 16.56 g/kg in the 1st year to 3.97 g/kg in the 9th year, since the accumulation rate is slower than the decomposition rate. The SOM content change has been tested in the mudflat reclamation area of Hangzhou Bay and at the mouth of the Yangtze River [37]. The sink velocity would exceed the decomposition velocity and the SOM content would increase; artificial soil improvement will also have tremendous effects on the SOM content.

**Fig 7:**
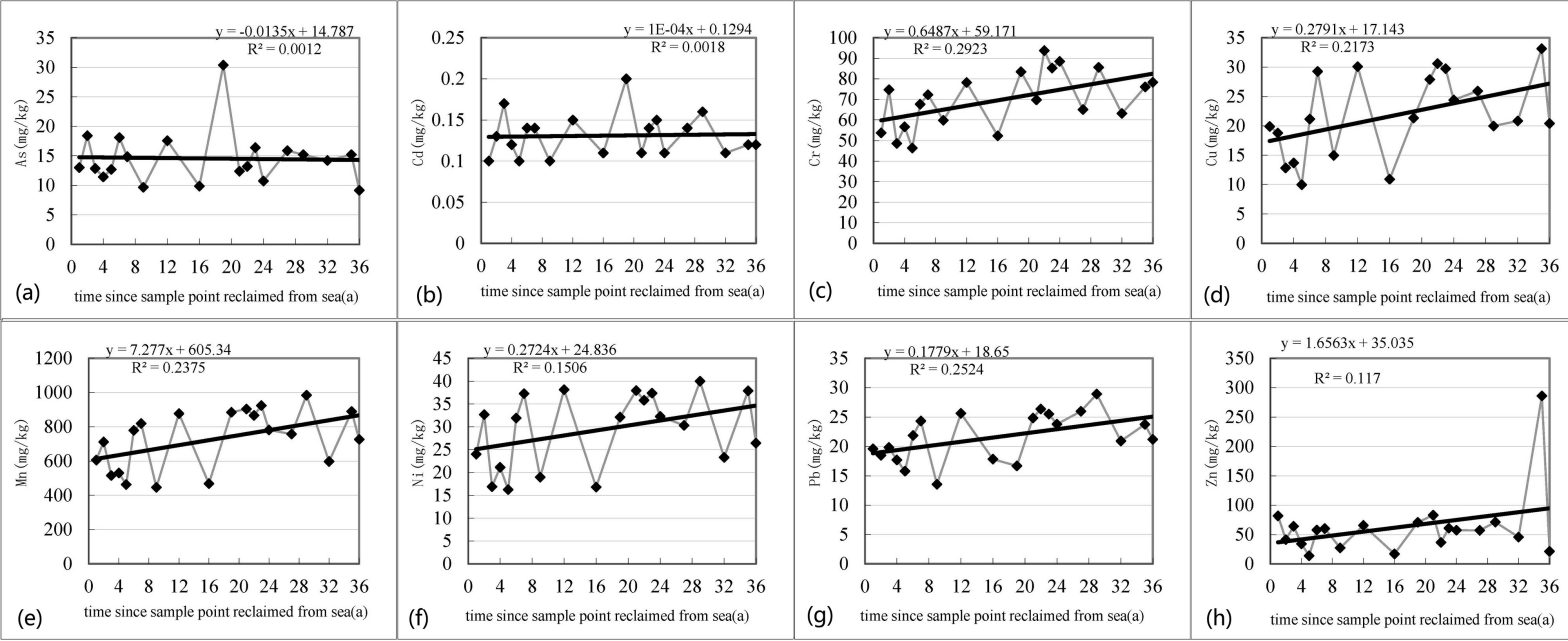
The soil heavy metal concentration change along the reclamation period. The elements of Cu, Pb, Ni, Cr, Zn, As, Cd, and Mn have a high correlation with each other. The Pb, Cr, Ni, Zn concentration exhibited an increasing trend over the 36 years. The Mn concentration appear as a growth trend during the 36-year reclamation period. The concentration demonstrated continuously decreased trend.

Heavy metal concentrations have a high correlation with each other, and the Cu concentration exhibited an increasing trend over the 36-year reclamation period (r = 0.47) and decreased from 19.88 mg/kg to 9.98 mg/kg in the first 5 years due to rainfall leaching. The Pb concentration exhibited an increasing trend over the 36 years (r = 0.50). It decreased from 19.56 mg/kg to 13.53 mg/kg in the first 9 years (Fig 7). The Cr concentration exhibited an increasing trend over the 36-year reclamation period (r = 0.54) and decreased from 53.8 mg/kg to 46.38 mg/kg. The Ni concentration exhibited an increasing trend (r = 0.39), but it decreased from 24 mg/kg to 16.23 mg/kg in the first 5 years. The Zn concentration exhibited an increasing trend during the 36-year reclamation period (r = 0.34) and decreased from 81.52 mg/kg to 13.72 mg/kg in the first 5 years. The concentration of As and Cd changed slightly. The Mn concentration appeared as a growth trend during the 36-year reclamation period (r = 0.49). It decreased from 605 mg/kg to 446 mg/kg in the first 9 years.

During coastal reclamation, most heavy metal elements demonstrated a growth trend, but showed decreases during the initial period. At the beginning period of reclamation, the area had not launched large-scale industrial construction and urbanization. Most of the heavy metal concentrations within the reclamation materials were transferred along with the precipitation leaching and made the heavy metal concentrations decrease continuously [38]. When the reclamation project reached the 5th-9th year, the on-going industrial and urban construction projects brought external source heavy metals and, therefore, contamination of surface land, which served as a sink. The sink velocity exceeded precipitation leaching and velocity, and soil heavy metal concentrations increased. Human activities caused Cu, Cr, Ni, and Mn accumulation during the initial 10 years, and Zn, Pb accumulated in the 10th-20th years. However, Cd and As did not accumulate, but the potential ecological risk should not be ignored.

## Discussion

The reclamation periods in China can be divided into four periods (Table 8). Reclaimed land used for agriculture and mariculture before 1990 is now mainly used for urban areas, ports, and coastal industry expansion driven by the rapid development of the coastal economy. Mudflats and offshore areas have experienced the reclamation process, and the coastal environment has consequently transformed along with the effects of natural processes and human activities. Three large-scale coastal reclamations occurred from 1950 to mid-1970, from mid-1970 to 1980, from 1980 to 1990, and from the 1990s-present [6]. During the 1950s and 1980s, the two large-scale reclamation coastal areas were used to expand the farmland area and develop harbour areas, accounting for 91.4% and 3.9% of the total reclaimed area, respectively, while other reclamation use types contributed to a small proportion [32]. Reclamation was adopted to develop salt production pans during the early stages of China’s national development, to accelerate agriculture in the 1960s and 1970s, and to enhance mariculture in the 1980s and 1990s [5]. However, new reclamation in the 21st century has been directed toward expanding harbour construction, coastal industrialization, petrochemical investments, and urbanization space, accounting for 42.5% of the total reclamation area, whereas reclamation for agricultural purposes decreased obviously to only 22.6%, and focus shifted to the development of megacities in the coastal and bay areas since the 2000 [1,11].

**Table 8:** Four stages of reclaimed land since 1949 in China.

Time	The main reclamation area	Utilization	Main investment/operation types	Representative engineer
1950s-1970s	In high and middle tidelands	Increasing salt, agricultural, and fishery production	By government and local farmers	Chongming Island, Shanghai; Changlu salt field
1970s-1980s	In middle and low tideland	Saline, marculture	By government and farmers	Shilihai farm of Tangshan, Hebei Province
1980s-2000s	Low tidelands, offshore area and bay	Mariculture, urban and industry	By Joint stock company	Jiaozhou Bay, Shandong Province
2000s-present	In mudflat, bay, inshore	Industrial, port, urban	By national investment	Tianjin Binhai New Area; Caofeidian, Hebei Province

Inspired by the fast-growing economy, local governments have aggressively expanded coastal industries and promoted urbanization, which has resulted in an increasing demand for land area through coastal reclamation [5, 9]. Coastal reclamation is highly efficient at solving the contradiction between land scarcity and land requirements for agriculture, industry, and urban development [6]. China has established strict farmland protection policies to define a 'red line' for farmland areas because the acceleration of urbanization at the expense of arable land threatens food supply and demand [5]. Coastal reclamation is defined as unused land that is easy to convert into built land and that provides tremendous benefit compared to occupied farmland that involves paying a farmland reclamation fee. While there is no strict protection policy governing coastal reclamation, it costs less to reclaim coastal areas than to occupy farmland for local governments. Therefore, China has experienced severe coastal land reclamation at fast speeds, over a large area, and to a broader extent in recent decades, which has resulted in a significant loss of coastal wetlands and wildlife habitats, along with the degradation of marine ecosystems [1,5,6,10].

Many previous research has conducted the effects of coastal reclamation upon wetlands ecosystems. Xiao et al. [39] found that heavy metals in ditch wetland accumulated with increasing reclamation history and Cd posed a medium to high environmental risk while low risk for other metals. Long reclamation history caused lower bioconcentration factor (BCF) and translocation factor (TFs) in ditch wetlands (DWs) and higher levels in riparian wetlands (RWs). Her findings is correlated with the research that the concentration of trace elements exhibited an increasing trend during the reclamation period. Bai et al. [40] collected the soil profile samples to a depth of 70 cm in both degraded wetland and freshwater restored wetlands in the Yellow River Delta of China to analyze the trace element pollution effects of freshwater input on coastal wetland soil. The enrichment factor (EF) values for Cu, Ni and Pb in both wetlands indicated minimal enrichment levels, whereas both As and Cd were significantly enriched with EF values 3 or 6 times greater than 1.5. As and Ni exceeded the effect range low and threshold effect level in both wetlands. Bai et al. [41] collected in three salt marshes with different plant species in the Yellow River Delta and founded the highest value for each sampling site was observed in summer and the lowest one in fall. Compared to other elements, both Cd and As had higher enrichment factors exceeding moderate enrichment levels. The toxic unit values of these trace elements did not exceed probable effect levels. The correlation analysis showed that these trace elements were closely linked to soil properties. In this study, we also found that the volume weight had a negative correlation with Cu, Pb, Mn, and Hg, showing that good soil structure might accumulate more heavy metal concentration.

Establishing of an ecological red line system is an effective policy tool for marine ecosystems and spatial resource management and would decrease the marine diversity loss, water quality deterioration offshore, and pertinent changes in coastal landscapes due to blind coastal reclamation [2]. There is a sea-land separation dilemma in land management and environmental protection. The 'blue line' regulation system, aimed at restraining decreased coastal wetland areas at a national, provincial or local level in important ecological function areas and in vulnerable areas, should be established as early as possible [2]. Making laws to suppress local government enthusiasm has been tested as an effective method in coastal China.

## Conclusion

This paper applied remote-sensing techniques to analyse the spatial and temporal distributions of coastal reclamation in Bohai Bay, China and to investigate the relationships between coastal land reclamation and soil property changes. Analysis of long-term Landsat remote sensing images from 1974 to 2010 in one-year intervals indicated a trend of land reclamation. Approximately 901.7 km^2^ offshore and mudflat areas have been turned into developed land in the past 36 years, which has led to the disappearance of 56.9% of mudflat area, 26.7% of the island area and 9.3% of offshore areas. The water content is relatively lower compared to natural salt lands (36.46%~40.89%) and newly reclaimed land, whereas the water content of the Binhai New Area was lower than that in the Caofeidian New Area. The salinity in the newly reclaimed areas was lower owing to the poor of retention of salinity in sandy soils. The Pb, Cr, Ni, Cd, and Mn concentrations were higher with a longer reclamation history, whereas Zn enrichment was found in the Nanjiang portal area and Caofeidian New Area. The average concentration of Mn was 686.91 mg/kg and the ascending order of heavy metal concentrations were Cr>Zn>As>Ni>Cu>Pb>Cd>Hg. Approximately 20.5% of the samples exceeded the Tianjin soil background value and 31.25% exceeded the Bohai Bay surface sediment background value. Heavy metal elements had an obvious positive correlation, except with Zn, and Cd, implying that they had a high spatial relationship and a high similarity of source. The coastal reclamation may have effects upon the concentration of heavy metals in the reclaimed coastal wetland.
